# Potential Ameliorative Effect of Aged Liubao Tea Aqueous Extract on D-Galactose-Induced Pathological Damage in an Alzheimer’s Disease Zebrafish Model

**DOI:** 10.3390/ph19091336

**Published:** 2026-08-24

**Authors:** Shini Zhang, Xiao-Li Luo, Hangxing Yang, Ganghua Huang, Jing Leng, Tao Yang

**Affiliations:** 1 School of Pharmaceutical Sciences, Guangxi University of Chinese Medicine, Nanning 530200, China; shiniz@163.com (S.Z.); lxiaoli2017@126.com (X.-L.L.);; 2 Teaching and Experimental Training Centre, Guangxi University of Chinese Medicine, Nanning 530200, China; 3 China Tea (Wuzhou) Co., Ltd., Wuzhou 543000, China; 4 Guangxi Key Lab High Incidence Infect Dis Integrat, Guangxi University of Chinese Medicine, Nanning 530200, China

**Keywords:** black tea, Alzheimer’s disease, zebrafish, pathological damage, active constituent

## Abstract

**Background:** Alzheimer’s disease (AD) is a prevalent neurodegenerative disorder for which effective treatments remain limited. Liubao tea, a distinctive post-fermented dark tea, develops unique aged and areca nut aromas through prolonged storage. However, comprehensive studies on its anti-Alzheimer’s disease effects are lacking. This study aimed to identify the bioactive constituents of Liubao tea with varying storage durations and aroma profiles, and to further evaluate its neuroprotective potential against Alzheimer’s disease. **Methods:** Chromatography and spectrophotometry were employed to quantitatively determine the primary bioactive components in various Liubao tea samples. A D-galactose-induced Alzheimer’s disease zebrafish (*Danio rerio*) model was established to evaluate the neuroprotective effects of the aqueous extract of Liubao tea. **Results:** The contents of theabrownins, theaflavins, tea pigments, and catechins showed potential associations with the anti-AD capacity of Liubao tea. Variations in these bioactive components appeared to correlate with the neuroprotective activity. **Conclusions:** This study elucidates the anti-AD potential of aged Liubao tea and associates its characteristic constituents with neuroprotective effects. The findings provide essential theoretical support for developing Liubao tea as a promising natural product candidate worthy of further anti-AD research.

## 1. Introduction

Tea, a natural dietary beverage enriched with polyphenols and a spectrum of bioactive constituents, possesses remarkable potential for the prevention and amelioration of Alzheimer’s disease (AD). Numerous studies demonstrate that polyphenols from green and black tea can mitigate oxidative stress and cholinergic deficits associated with AD pathology, although neuroprotective potency varies substantially across different tea types and chemical fractions [1,2]. Owing to variations in processing methods, different tea varieties, including green, white, yellow, oolong, black, and dark tea, exhibit unique chemical profiles, which contribute to distinct and complementary neuroprotective effects. In green tea, epigallocatechin gallate (EGCG) is the primary bioactive compound responsible for Amyloid-β (Aβ) aggregation and mitigating oxidative stress as well as neuro-inflammation [3,4]. Accumulating comparative evidence reveals that white and yellow teas possess neuroprotective and anti-Aβ properties comparable to those of green tea and, in some cases, exhibit even more potent effects [5,6]. Oolong tea extracts primarily ameliorate cognitive and behavioral deficits in AD animal models by modulating neuro-inflammation and correcting metabolic disturbances [7,8]. Aqueous extracts of black tea significantly prolonged the lifespan of nematodes in the *Caenorhabditis elegans* (*C. elegans*) model. Moreover, these extracts substantially delayed Aβ-triggered progressive paralysis, alleviated pathological symptoms in the AD nematode model, and enhanced the oxidative stress tolerance of *C. elegans* under chromium-induced oxidative damage [9]. Epidemiological evidence suggests that habitual consumption of dark tea is associated with a reduced risk of age-related cognitive decline in humans. Additionally, accumulating preclinical studies indicate that dark tea extracts exhibit stronger anti-aging and anti-amyloid effects than green tea in rodent and invertebrate models. Liubao (Liupao) tea and Pu-erh tea are typical representatives of Chinese dark teas. During post-fermentation and long-term aging, catechins are progressively transformed into theaflavins (TFs), thearubigins (TRs), and theabrownins (TBs). TFs and TRs can inhibit Aβ fibrillization and alleviate microglial inflammation, whereas TBs, the characteristic polymeric pigments unique to dark tea, have been recognized as key bioactive components that improve cognitive function by maintaining gut homeostasis and suppressing neuro-inflammatory cascades [10,11]. Current research on the anti-Aβ properties of dark teas has primarily focused on Pu-erh tea, which has been shown to mitigate Aβ toxicity by modulating conserved signaling pathways in *Caenorhabditis elegans* models [12]. In contrast, studies investigating the anti-AD effects of Liubao tea, which is renowned for its unique Aged aroma (Chen Xiang) and Betel nut aroma (Bing-lang Xiang), remain scarce. Furthermore, it remains unclear whether dynamic changes in bioactive constituents during the long-term aging of Liubao tea correlate with variations in its neuroprotective activity.

Zebrafish (*Danio rerio*) serve as ideal vertebrate models for exploring AD pathogenesis and therapeutic interventions due to their high genetic conservation, embryonic optical transparency, and suitability for high-throughput screening [13]. For in vivo functional verification, the neuroprotective potential of Liubao tea was assessed using a well-established D-galactose-induced AD-like model, which accurately recapitulates the core pathological hallmarks of AD, including excessive oxidative stress, persistent neuro-inflammation, and significant cognitive dysfunction. Compared to mammalian models, zebrafish lack the layered and complex cerebral cortex found in humans, and their neurodegenerative progression does not fully recapitulate the pathological characteristics of sporadic Alzheimer’s disease in humans. Furthermore, larval zebrafish have an immature blood–brain barrier, which differs structurally and functionally from that of adult mammals.

This study aims to investigate the neuroprotective effects of Liubao tea with different aging periods and characteristic aroma types against Alzheimer’s disease (AD). An integrated strategy combining phytochemical profiling and in vivo bioevaluation was employed. Specifically, seven major bioactive constituents (catechins, total polyphenols, free amino acids, proanthocyanidins, theaflavins, thearubigins, and theabrownins) in Liubao tea samples across six aging gradients (unaged to 15 years) and two aroma types (Aged aroma and Betel nut aroma) were quantified by high-performance liquid chromatography (HPLC). Neuroprotective activities were subsequently evaluated in a D-galactose-induced AD-like zebrafish model, with endpoints including locomotor function, memory capacity, redox homeostasis, and pro-inflammatory cytokine levels. Finally, correlation analysis was conducted to elucidate the relationship between dynamic compositional variations and neuroprotective efficacy.

## 2. Results

### 2.1. Characteristic Chemical Compositions of Liubao Tea

**Figure 1:**
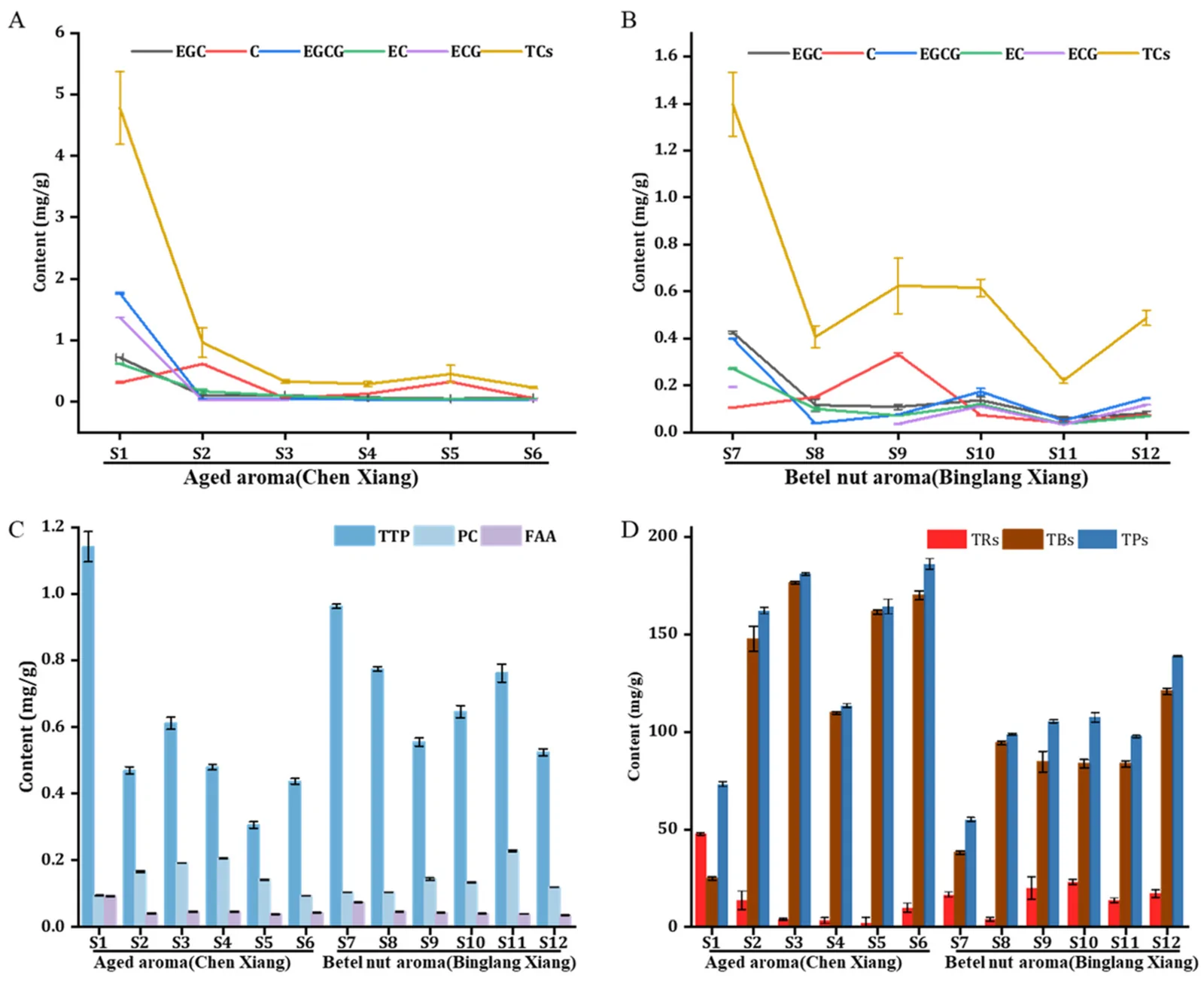
HPLC-measured catechin content variations in Aged aroma (**A**) and Betel nut aroma Liubao tea (**B**) across different aging years. The dynamic changes in characteristic chemical components ((**C**): TTP, PC, FAA; (**D**): TRs, TBs, TPs) of Liubao tea during the aging process.

Characteristic chemical components in Liubao tea water extract include Catechins (EGC, C, EGCG, EC, ECG), CAF, TP, FAA, PC, TFs, TRs and TBs. The HPLC validation data were established and evaluated (Table S1, Supplementary Materials). Chromatograms of the Liubao tea extract and the standard mixture are shown in Figures S3–S5 (Supplementary Materials). Catechin levels in aged Liubao tea (**S2**–**S6**, **S8**–**S12**) were markedly lower than those in unaged samples **S1** and **S7** (Figure 1A,B, and Table S2), suggesting the transformation of catechins into tea pigments.

The total catechin content decreased from 4.78 to 0.23 mg·g^−1^ in Aged aroma Liubao tea (**S1**–**S6**) and from 1.40 to 0.49 mg·g^−1^ in Betel nut aroma Liubao tea (**S7**–**S12**). Catechin content in S8 is not detected (Figure 1B). The catechin in the Aged aroma type declined by approximately 20.78-fold, which is substantially greater than the 2.86-fold reduction observed in the Betel nut aroma type, indicating that catechins degrade much more rapidly during aging in the Aged aroma type. However, caffeine content remained nearly unchanged during aging, indicating that the aging process barely affected caffeine levels (Table S2, Supplementary Materials).

The total polyphenol content of Liubao tea exhibited a decreasing trend with prolonged aging. The initial polyphenol content was highest in the unaged samples, with the Aged aroma Liubao tea reaching 1.15 mg·g^−1^ (**S1**) and the Betel nut aroma Liubao tea reaching 1.51 mg·g^−1^ (**S11**). The free amino acid content showed an overall trend of first increasing and then decreasing during natural aging. The proanthocyanidin content remained relatively stable throughout the aging process, with only negligible changes observed as aging years increased. Specifically, the proanthocyanidin content of the Aged aroma type (**S1**–**S6**) ranged from 0.04 to 0.09–0.04 mg·g^−1^ for Aged aroma type (**S1**–**S6**), while that of the Betel nut aroma type (**S7**–**S12**) varied slightly from 0.07 to 0.04 mg·g^−1^ (Figure 1C and Table S3).

Tea pigments are the predominant characteristic components of Liubao tea, primarily comprising theaflavins (TFs), thearubigins (TRs), and theabrownins (TBs). Among these fractions, TFs were present at the lowest levels across all samples, with contents ranging from 0.35 to 2.52 mg·g^−1^ in samples **S1**–**S12**. TRs constituted the second most abundant tea pigment fraction and exhibited a distinct declining trend during the aging of Aged aroma Liubao tea (**S1**–**S6**), decreasing markedly from 47.56 to 9.67 mg·g^−1^; in contrast, TRs content remained relatively stable throughout the aging process in Betel nut aroma Liubao tea (**S7**–**S12**). As the most dominant pigment component, TBs showed a continuous increasing trend with extended aging. Specifically, TBs content increased from 24.83 to 170.0 mg·g^−1^ in Aged aroma Liubao tea (**S1**–**S6**) and rose from 38.07 to 120.77 mg·g^−1^ in Betel nut aroma Liubao tea (**S7**–**S12**). Notably, the TBs content of the Aged aroma type increased approximately 6.85-fold, which was substantially higher than the 3.17-fold increase observed in the Betel nut aroma type, demonstrating that TBs accumulation was more pronounced in Aged aroma Liubao tea during aging. Correspondingly, the total tea pigment content gradually increased with prolonged aging duration, peaking in the 15-year-aged tea samples. The maximum total tea pigment contents reached 186.05 mg·g^−1^ for the Aged aroma type (**S6**) and 138.62 mg·g^−1^ for the Betel nut aroma type (**S12**), respectively (Figure 1D and Table S3). Variations in the chemical components of Liubao tea among samples with different aging durations are largely attributable to the metabolic activities of fermentation microbes. In this study, only Liubao tea samples with fixed aging periods were examined, and dynamic alterations in bioactive constituents during long-term aging were not systematically evaluated. Prolonged storage can induce continuous oxidation, polymerization, and degradation of phenolic compounds, thereby altering the chemical profile and biological activity of Liubao tea.

To investigate the ameliorative effects of Liubao tea on pathological injuries in a D-galactose-induced zebrafish AD-like model, behavioral tests were conducted first. Subsequently, typical pathological features of AD, including senescent cell accumulation and Aβ protein aggregation, were thoroughly examined. Finally, levels of oxidative stress, cholinergic function, and inflammatory cytokines were measured to elucidate the underlying regulatory mechanisms.

### 2.2. Effect of Liubao Tea on Motor Impairment of Zebrafish Larvae

**Figure 2:**
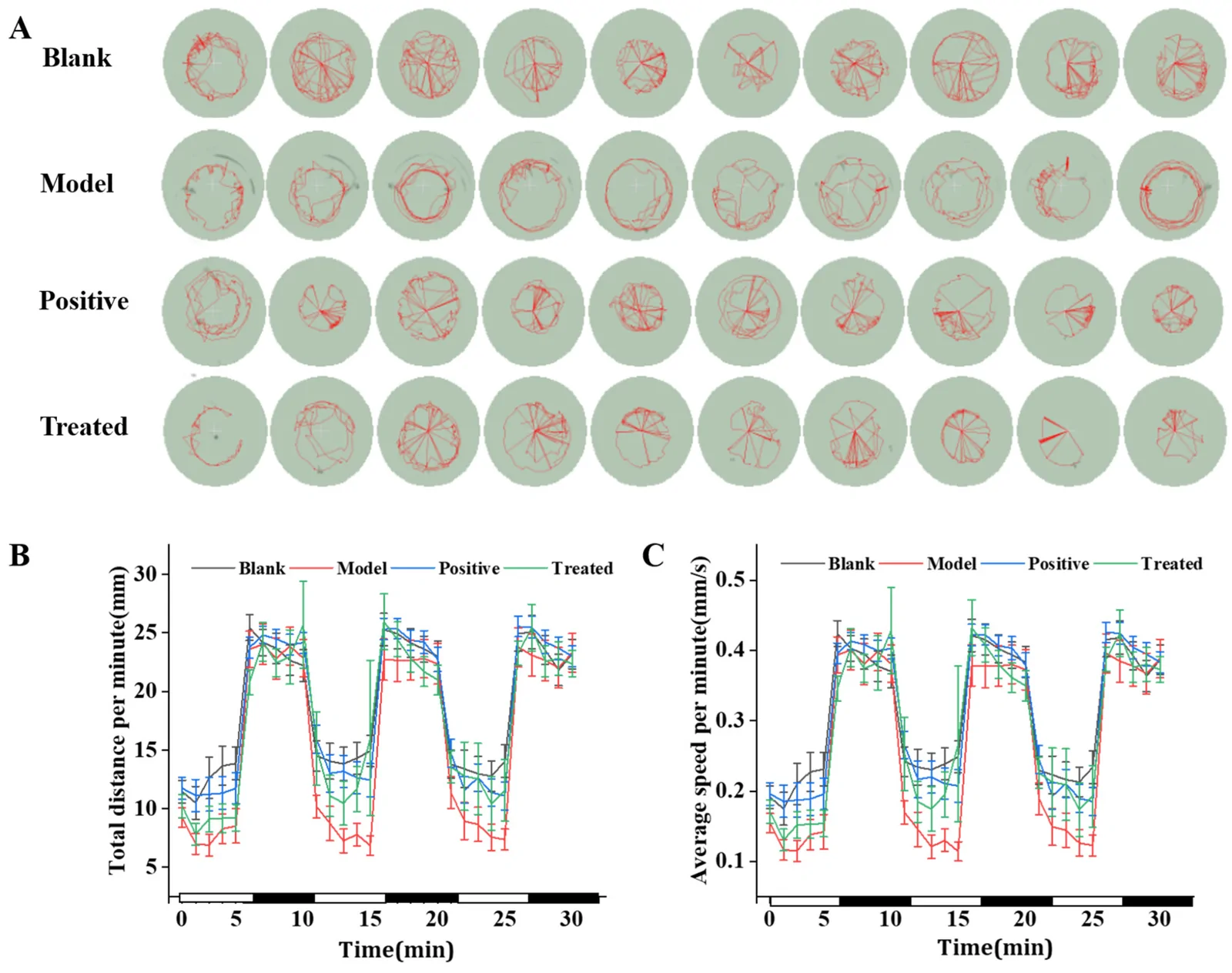
Effects of Liubao tea on motor impairment in AD zebrafish larvae. (**A**) Typical motion path of zebrafish after Liubao tea treatment. Total moving distance (**B**) and average movement speed per minute (**C**) after Liubao tea treatment. Note: The black and white segments on the abscissa represent the light–dark alternation period: black indicates dark treatment, and white indicates light treatment.

Decline in motor ability is a hallmark clinical symptom of AD. A behavioral analyzer was used to assess the regulatory effect of Liubao tea on locomotor function in zebrafish. Impaired responsiveness is a common clinical feature of AD patients. A light–dark alternation behavioral assay was conducted to evaluate the sample-induced improvement in the recovery capacity of AD zebrafish to stress stimuli. Typical motion paths of zebrafish are shown in Figure 2A. Total moving distance and average movement speed per minute are presented in Figure 2B and Figure 2C, respectively. As shown in Figure 2C, the average swimming speed per minute of zebrafish in the model group was lower than that in all other groups.

**Figure 3:**
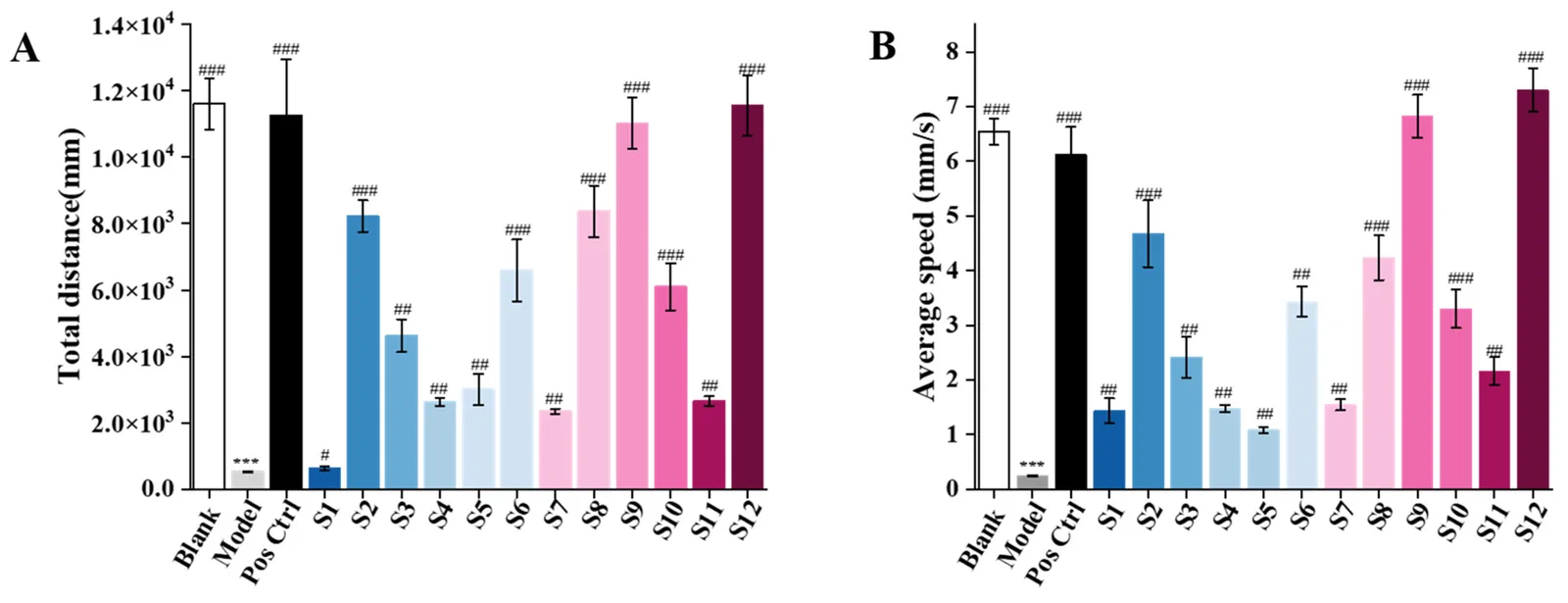
Total moving distance (**A**) and average movement speed per minute (**B**) of Blank, Model, Positive and after Liubao tea treatment group (**S1**–**S12**). Note: *** *p* < 0.001; vs. model group, ^#^ *p* < 0.05, ^##^ *p* < 0.01, ^###^ *p* < 0.001, *N* = 10.

Both the total swimming distance of zebrafish (Figure 3A) and the average light–dark speed per minute (Figure 3B) in the model group were significantly decreased compared with the blank control group (highly significant, *p* < 0.001), indicating successful model establishment. Compared with the model group, both the Aged aroma type (**S1**–**S6**) and Betel nut aroma type (**S7**–**S12**) significantly increased the total locomotor distance of zebrafish. The maximum total locomotor distance for the Aged aroma type was 8228.27 mm (**S2**), while that of the Betel nut aroma type reached 11,555.78 mm (**S12**), representing increases of 15.63-fold and 21.95-fold over the model group, respectively. The positive control group showed a 21.40-fold increase. These results indicate that both the Aged aroma type (**S1**–**S6**) and Betel nut aroma type (**S7**–**S12**) effectively ameliorated locomotor dysfunction. Similarly, compared with the model control group, both the Aged aroma type (**S1**–**S6**) and Betel nut aroma type (**S7**–**S12**) significantly increased the average light–dark locomotor speed per minute of zebrafish. Among them, **S2** and **S12** showed the most pronounced effects, with speeds increased by 18.64-fold and 29.08-fold, respectively, while the positive control group increased by 24.40-fold. These findings suggest that both the Aged aroma type (**S1**–**S6**) and Betel nut aroma type (**S7**–**S12**) of Liubao tea can effectively ameliorate responsiveness impairment in zebrafish.

### 2.3. Detection of SA-β-Gal Activity and Aβ Protein Aggregation in Zebrafish Larvae

**Figure 4:**
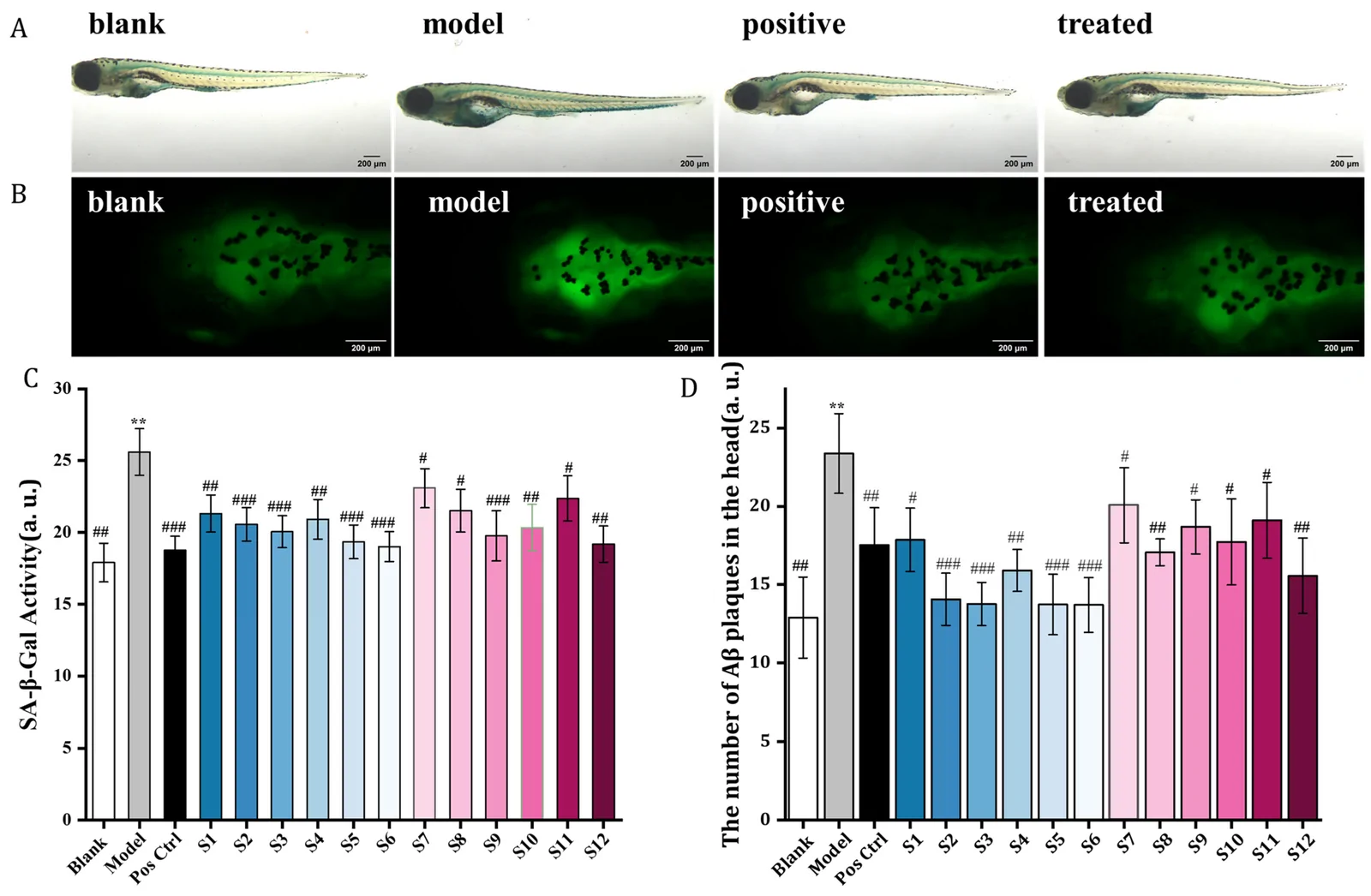
Representative photographs (**A**) and immunofluorescence images (**B**) of zebrafish AD-like model; SA-β-Gal activity (**C**) and the number of Aβ plaques in the zebrafish brain (**D**). Note: ** *p* < 0.01; vs. model group, # *p* < 0.05, ## *p* < 0.01, ### *p* < 0.001, *N* = 10.

Senescence-associated β-galactosidase (SA-β-Gal) is a well-established biomarker for cellular senescence. Consequently, the level of cellular senescence can be quantitatively evaluated by measuring intracellular SA-β-Gal activity. Positive blue staining was observed in zebrafish tissues following histological staining. Compared to the blank control group, the model group exhibited significantly intensified blue staining, indicating that D-galactose induction increased the abundance of senescent cells and triggered a robust senescence response in zebrafish (Figure 4A,B and Figure S6A). In contrast, treatment with Aged aroma (**S1**–**S6**) and Betel nut aroma (**S7**–**S12**) Liubao tea markedly reduced the intensity of blue staining. These results suggest that Liubao tea significantly decreased the number of senescent cells, thereby alleviating the aging phenotype. Consistently, SA-β-Gal activity was significantly upregulated in the model group compared to the blank control group (*p* < 0.01), confirming the induction of cellular senescence. Relative to the model group, the positive control group and the Liubao tea treatment groups (Aged aroma (**S1**–**S6**) and Betel nut aroma (**S7**–**S12**), presented distinct reductions in SA-β-Gal activity, mitigating senescence-related pathological changes. Among all samples, Aged aroma Liubao tea (**S6**) and Betel nut aroma Liubao tea (**S12**) exhibited the most potent ameliorative effects (Figure 4C).

Aβ aggregation and cerebral deposition are well-established pathological hallmarks driving the progression of AD. To further evaluate the anti-AD efficacy of Liubao tea, thioflavin S fluorescence staining combined with ImageJ quantitative analysis was performed to assess cerebral Aβ deposition in zebrafish larvae.

As shown in Figure 3B and Figure S6B, the model group exhibited strong fluorescence signals in larval brain tissues, indicating severe Aβ aggregation and extensive pathological deposition. Notably, treatment with the positive control drug, and Aged aroma (**S1**–**S6**) and Betel nut aroma (**S7**–**S12**) Liubao tea, effectively reduced cerebral Aβ fluorescence intensity in zebrafish larvae. Moreover, samples **S6** and **S12** demonstrated superior protective effects compared to the other tea samples. Quantitative analysis further confirmed that the model group had a significantly increased number of cerebral Aβ plaques relative to the blank control group (*p* < 0.001). In contrast, both the positive control drug and Liubao tea interventions significantly decreased Aβ plaque deposition in zebrafish brains. Collectively, these results demonstrate that Liubao tea exerts prominent anti-AD pharmacological effects in the D-galactose-induced zebrafish AD-like model. Its protective efficacy against AD pathological lesions is primarily attributed to the suppression of cellular senescence and the mitigation of cerebral Aβ deposition.

### 2.4. Effects of Liubao Tea Aqueous Extracts on ROS, SOD, MDA, AChE, IL-6 and TNF-α Levels in Zebrafish

**Figure 5:**
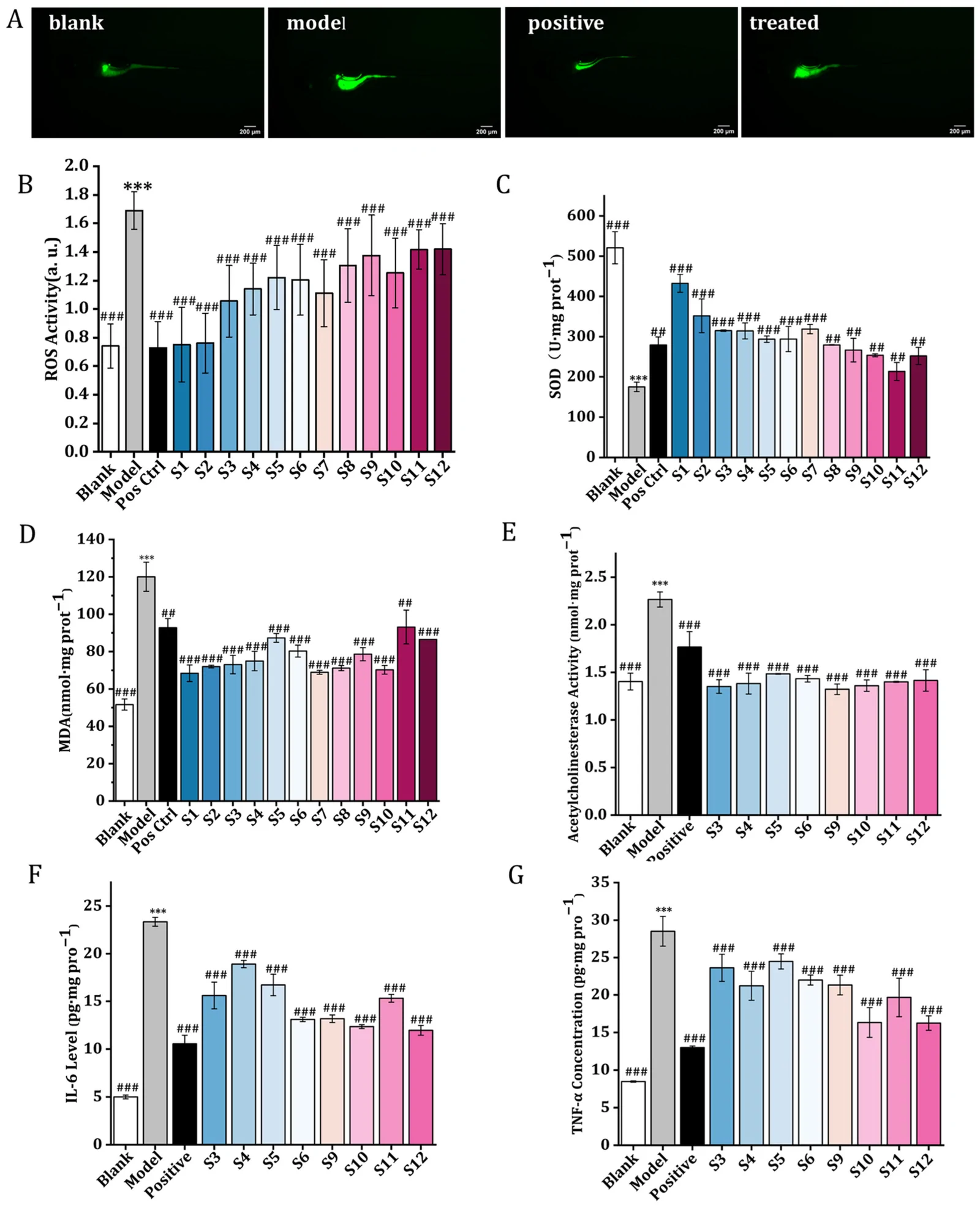
(**A**) Representative immunofluorescence images of ROS expression; (**B**) ROS fluorescence intensity; (**C**) SOD activity; (**D**) MDA content; (**E**) AChE activity; (**F**) IL-6 level; (**G**) TNF-α level. Compared with the blank control group. Note: *** *p* < 0.001; compared with the model group, ^##^ *p* < 0.01, ^###^ *p* < 0.001, *N* = 90.

Oxidative stress serves as a critical upstream pathological trigger that induces neuronal oxidative damage and aggravates AD-like pathological phenotypes in D-galactose-exposed zebrafish. The model group exhibited significantly higher ROS fluorescence intensity compared to all Liubao tea treatment groups (Figure 5A), confirming that continuous D-galactose exposure caused excessive ROS production and severe oxidative stress damage in zebrafish larvae. Administration of Liubao tea aqueous extracts effectively suppressed this excessive ROS generation, with the unaged Liubao tea groups (**S1** and **S7**) demonstrating the strongest ROS-scavenging activity among all tested samples (Figure 5B and Figure S7, Supplementary Materials).

Quantitative analysis of antioxidant indicators revealed that zebrafish in the blank control group maintained normal antioxidant status, with SOD activity at 520.95 U·mg prot^−1^ and MDA content at 51.63 nmol·mg prot^−1^. Induction with D-galactose caused severe oxidative stress in the model group, as demonstrated by a sharp decline in SOD activity to 175.09 U·mg prot^−1^ and a significant increase in MDA content to 120.05 nmol·mg prot^−1^. These changes indicate depletion of endogenous antioxidant defenses and substantial lipid peroxidation damage. Intervention with Liubao tea (**S1**–**S12**) mitigated ameliorated D-galactose-induced oxidative stress injury in zebrafish, with unaged Liubao tea (**S1** and **S7**) showing the most pronounced protective effects. Following treatment, SOD activities in the **S1** and **S7** groups recovered to 432.44 U·mg prot^−1^ and 318.65 U·mg prot^−1^, respectively, significantly improving antioxidant capacity. Concurrently, their MDA levels decreased to 68.43 nmol·mg prot^−1^ and 68.93 nmol·mg prot^−1^, markedly reducing lipid peroxidation damage (Figure 5C,D). Overall, all Liubao tea samples (**S1**–**S12**) could effectively antagonize D-galactose-triggered oxidative stress damage by upregulating antioxidant enzyme activity and reducing lipid peroxidation product accumulation, thereby exerting reliable antioxidant neuroprotective effects.

Cholinergic dysfunction is a well-established pathological hallmark of AD and is closely associated with cognitive impairment. The model group exhibited significantly elevated AChE activity (2.267 nmol·mg prot^−1^) compared to the blank control group (1.404 nmol·mg prot^−1^, *p* < 0.001) (Figure 5E). This excessive AChE activity indicates severe impairment of cholinergic neurotransmission in the model zebrafish. The positive control drug, donepezil, significantly reversed these cholinergic abnormalities (*p* < 0.001), further validating the reliability of the established zebrafish AD-like model. Notably, all aged Liubao tea samples (**S3**–**S6**, **S9**–**S12**) significantly inhibited the abnormal activation of AChE (*p* < 0.001), with Aged aroma Liubao tea **S6** and Betel nut aroma Liubao tea **S10** demonstrating the most prominent efficacy. Specifically, **S6** treatment reduced AChE activity to 1.433 nmol·mg prot^−1^, while **S11** decreased AChE activity to 1.400 nmol·mg prot^−1^. These results demonstrate that aged Liubao tea effectively ameliorates cholinergic neurotransmission deficits via AChE inhibition, thereby alleviating AD-related cholinergic dysfunction.

Neuro-inflammation serves as a critical pathological driver that synergizes with oxidative stress and cholinergic damage to accelerate the progression of AD. Compared to the blank control group, the model group exhibited a significant upregulation of pro-inflammatory cytokines, with IL-6 increasing to 23.370 pg·mg prot^−1^ and TNF-α rising to 28.517 pg·mg prot^−1^, indicating robust neuro-inflammatory activation. In contrast, both donepezil and Liubao tea intervention significantly suppressed neuro-inflammatory responses (Figure 5F,G). Consistent with their cholinergic improvement effects, Aged aroma Liubao tea **S6** and Betel nut aroma Liubao tea **S12** demonstrated the most potent anti-inflammatory effects among all aged tea samples. The **S6** treatment notably reduced IL-6 and TNF-α levels to 13.097 and 21.989 pg·mg prot^−1^, respectively, while **S12** significantly decreased IL-6 and TNF-α levels to 11.963 and 16.245 pg·mg prot^−1^. Collectively, these findings confirm that aged Liubao tea effectively mitigates excessive neuro-inflammation in a D-galactose-induced zebrafish AD-like model by downregulating the expression of key pro-inflammatory mediators (TNF-α and IL-6). Among the samples **S6** and **S12** showing superior anti-neuro-inflammatory capacity, thereby slowing AD pathological deterioration.

### 2.5. Correlation Between Chemical Components of Liubao Tea Aqueous Extract and Neuropathological Characteristics in Zebrafish AD-like Model

**Figure 6:**
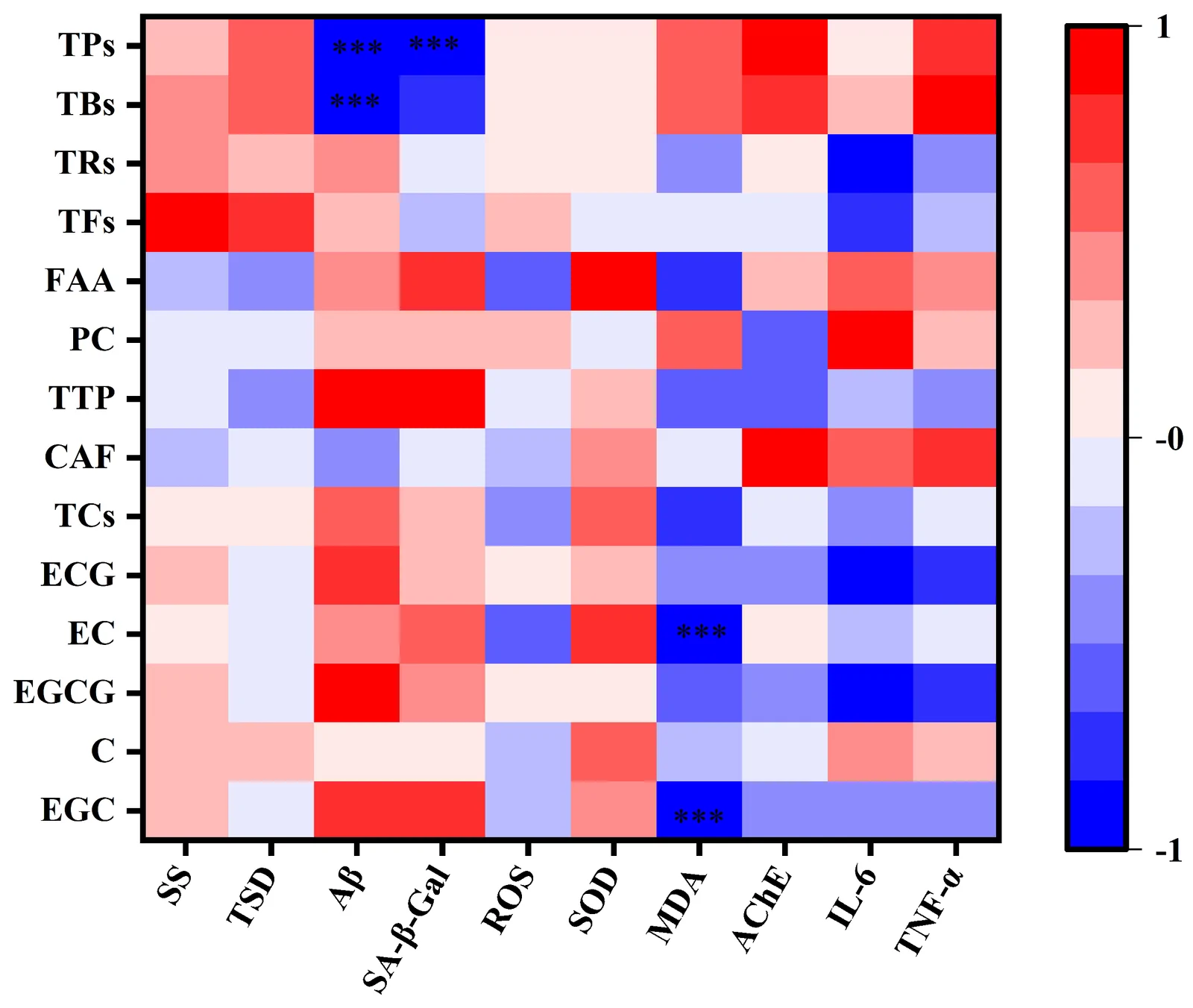
Spearman correlation heatmap between tea phytochemicals and AD-related indicators. Asterisks indicate statistically significant correlations after Benjamini–Hochberg FDR multiple-testing correction: *** FDR-*p* < 0.001.

Spearman’s rank correlation analysis was conducted to investigate the potential relationships between the bioactive components of Liubao tea and various physiological and pathological indicators. All pairwise comparisons were subjected to Benjamini–Hochberg false discovery rate (FDR) multiple-testing correction to minimize false-positive results arising from extensive statistical comparisons. The correlation outcomes before and after correction are summarized in Figure 6, and Tables S4 and S5. Behavioral tests demonstrated that aqueous extracts of Liubao tea significantly ameliorated locomotor and cognitive impairments in a zebrafish AD-like model. Specifically, TFs appeared to be positively associated with the total swimming distance (r = 0.73, raw *p* = 0.01) and swimming speed (r = 0.62, raw *p* = 0.03). However, these associations lost statistical significance after FDR correction (corrected (*p* = 0.11) and (0.21), respectively). Other components (ECG, TRs, TBs) including positive but non-significant correlation trends suggest that TFs may serve as potential bioactive substances mediating the cognitive-protective effects of Liubao tea. Consistent with these findings, sample **S9** (3-year-aged Betel nut areca Liubao tea) contained the highest TF content and produced the most pronounced cognitive improvement in the zebrafish models.

Mechanistically, TFs preserve neuronal integrity by scavenging excess ROS, suppressing oxidative stress and neuro-inflammation, and alleviating neuronal damage induced by abnormal protein aggregation [14]. Furthermore, catechin (C), EGCG, and ECG act as pivotal antioxidant and neuroprotective constituents, which can synergistically enhance the bioactivities of TFs by modulating downstream signaling cascades [15].

Regarding of neuronal senescence and Aβ pathological lesions, polymeric tea pigments were identified as the primary protective components. Specifically, TBs showed significant negative correlations with Aβ deposition (r = −0.92, *p* < 0.01) and SA-β-Gal activity (r = −0.71, raw *p* < 0.05), while TPs exhibited highly significant negative correlations with Aβ levels (r = −0.94, raw *p* < 0.01) and SA-β-Gal activity (r = −0.81, raw *p* < 0.01). It should be noted that several of these associations lost statistical significance after Benjamini–Hochberg FDR correction for multiple comparisons. In contrast, monomeric catechins (EGC, EGCG) were positively correlated with Aβ and SA-β-Gal levels. These findings further suggest indications that tea pigments, rather than catechin monomers, may serve as potential bioactive substances involved in inhibiting neuronal senescence and Aβ aggregation.

For oxidative stress modulation, EGC and EC exhibited highly significant negative correlations with MDA content (r = −0.90, raw *p* < 0.01; r = −0.94, raw *p* < 0.01), while TCs and FAA also negatively influenced MDA levels (raw *p* < 0.05). However, some of these correlations did not remain significant after FDR adjustment. These findings suggest that phenolic compounds and amino acids may help alleviate lipid peroxidation damage. Additionally, FAA was positively correlated with SOD activity (r = 0.69, raw *p* < 0.01), pointing a potential association with enhanced endogenous antioxidant capacity. Distinct regulatory effects on neurological function were also observed among tea components: caffeine (CAF) was positively correlated with AChE activity (r = 0.71, raw *p* < 0.05), whereas major catechins (EGCG, ECG) presented negative regulatory trends, reflecting the functional diversity of different bioactive components in Liubao tea.

In terms of neuro-inflammatory regulation, EGCG and ECG significantly decreased the levels of the pro-inflammatory cytokines IL-6 and TNF-α (raw *p* < 0.05). Furthermore, TFs and TRs showed strong negative correlations with IL-6 (r = −0.79, raw *p* < 0.05; r = −0.83, raw *p* < 0.05); however, some of these associations were no longer statistically significant after FDR correction. In contrast, TBs and TPs exhibited mild positive correlations with inflammatory factors. Liubao tea extract showed potential associations with both oxidative biomarkers and inflammatory factors. Based on the current data, we cannot determine which mechanism acts as the exclusive primary pathway. We propose that the neuroprotective effect arises from the simultaneous modulation of multiple interconnected pathways.

## 3. Discussion

This study demonstrated that Liubao tea, characterized by distinct aroma types and varying aging periods, can ameliorate D-galactose-induced AD-like pathological damage in zebrafish through multiple regulatory pathways (Table 1).

Swimming speed (SS) and total swimming distance (TSD) were used to evaluate the motor capabilities of zebrafish. Both the Aged aroma (**S1**–**S6**) and Betel nut aroma (**S7**–**S12**) type Liubao tea group exhibited superior motor performance compared to the model group. Among them, **S12**, the 15-year-aged Betel nut aroma sample, demonstrated the optimal motor performance. Regarding typical AD pathological characteristics, including cellular SA-β-Gal activity and aberrant Aβ protein aggregation, the 15-year-old Aged aroma Liubao tea (**S6**) showed the most remarkable protective effects. In terms of oxidative stress modulation, the model group displayed significant antioxidant system dysfunction, characterized by excessive ROS accumulation, reduced SOD activity and increased MDA level. The unaged Aged aroma Liubao tea (**S1**) exerted the most effective antioxidant effect by scavenging excess free radicals and restoring endogenous antioxidant capacity. Regarding cholinergic function regulation, **S6** effectively reversed the D-galactose-induced abnormal upregulation of AChE activity. For neuro-inflammatory modulation, **S12** markedly reduced IL-6 levels and downregulated TNF-α expression, thereby alleviating central neuro-inflammatory injury. Behavioral results demonstrated that Liubao tea aqueous extracts effectively ameliorated zebrafish locomotor deficits and cognitive impairments, which were closely correlated with the characteristic bioactive components of Liubao tea. Spearman’s correlation analysis suggested TFs are candidate functional substances potentially linked to behavioral improvement, with positive correlations observed between TF contents and zebrafish swimming distance and speed. Consistently, **S9** (3-year-aged Betel nut aroma Liubao tea), which had the highest TF content, showed the most prominent cognitive protective effect. Importantly, these statistical associations reflect correlation rather than direct causation. Further validation experiments using purified individual compounds or enriched fractions will be conducted in our follow-up research to confirm these findings.

Mechanistic analysis suggested that different components of Liubao tea may exert targeted and synergistic neuroprotective effects through multiple pathological pathways. Polymeric tea pigments (TBs and TPs) were identified as the dominant components potentially associated with the inhibition of neuronal senescence and Aβ aggregation, showing negative associations with Aβ deposition and SA-β-Gal activity. In contrast, monomeric catechins and free amino acids primarily contributed to antioxidant protection; for example, EGC and EC were associated with reduced MDA accumulation, while FAA correlated with increased SOD activity, potentially aiding the restoration of endogenous antioxidant capacity and alleviating lipid peroxidation damage. Moreover, phenolic monomers and TFs were linked to significant anti-neuro-inflammatory effects, suggesting a potential role in downregulating pro-inflammatory cytokines IL-6 and TNF-α. Conversely, tea pigments exhibited weaker associations with inflammatory markers, indicating divergent functional roles among different constituent classes. In this study, the extract was associated with the simultaneous suppression of oxidative stress and Aβ deposition. We acknowledge that only Spearman correlation analysis was performed in the current work. This method alone cannot definitively identify TFs, TBs, and tea pigments as the core active substances. At this stage, we cannot confirm a direct binding interaction between Liubao tea extract and the Aβ peptide. Further in vitro molecular experiments will be conducted to verify whether the purified compounds or extract directly inhibit Aβ aggregation.

Epidemiological studies have confirmed that long-term tea consumption is significantly associated with a lower incidence of AD [16]. Tea catechins and theanine act as potent free radical scavengers that penetrate the blood–brain barrier and suppress lipid peroxidation through multiple mechanisms, including direct scavenging of excess free radicals, interruption of free radical chain reactions, activation of intracellular antioxidant enzymes, and chelation of iron and copper ions. These actions ultimately reduce the accumulation of MDA and other lipid peroxidation products [17]. Consistent with these findings, our study demonstrated that tea pigments, FAA, and catechin components in Liubao tea were negatively correlated with ROS and MDA levels and positively correlated with SOD activity (all *p* < 0.05). These results suggested that the aforementioned bioactive components alleviate oxidative stress, thereby modulating Aβ deposition and the senescence-associated secretory phenotype. Moreover, EGCG has been shown to cross the blood–brain barrier, upregulate SOD and catalase activities, mitigate oxidative stress in AD models, and exert anti-inflammatory effects by modulating microglia-induced cytotoxicity and activating neuronal survival-related signaling pathways [18]. Regarding polymeric pigments such as TFs and TBs, evidence concerning their blood–brain barrier permeability remains inconsistent. Most high-molecular-weight theabrownins may have limited brain penetration; their in vivo neuroprotective effects could partially depend on peripheral regulation, gut–brain axis signaling, or metabolite-mediated mechanisms, as discussed in recent studies on fermented tea.

Previous studies have clearly demonstrated that TFs modulate the cholinergic system by inhibiting AChE activity, thereby enhancing cholinergic function [19]. Consistent with our current results, TFs may potentiate the anti-AD effects of Liubao tea through two intersecting mechanisms: anti-inflammatory modulation and cholinergic regulation. Additionally, emerging evidence indicates that TFs alleviate aging-induced cognitive dysfunction via the microbiota–gut–brain axis, providing a novel mechanistic explanation for the significant negative correlation between TFs and the senescence marker SA-β-Gal observed in this study. Structurally, TFs can be progressively oxidized and polymerized into TRs, which serve as key antioxidants defending against biological oxidation during aging. TRs are further oxidized and polymerized into TBs. As natural macromolecular constituents, TBs exert prominent anti-aging properties by regulating lipid, energy, and amino acid metabolism [20]. Aged tea samples exhibited stronger anti-inflammatory and cholinergic effects, whereas unaged tea demonstrated higher antioxidant activity. This phenomenon can be explained by the reduction in phenolic hydroxyl groups. As monomeric phenols polymerize into polymeric pigments during aging, the contents of TRs and TBs increase correspondingly. Variations in processing methods, microbial communities during post-fermentation, and storage conditions further shape the chemical profiles of the Aged aroma and Betel nut aroma subtypes, accounting for inter-sample differences in neuroprotective efficacy. In this study, we quantified only several representative polyphenols. Numerous low-abundance compounds remain unidentified and may also participate in neuroprotective effects.

Overall, this study demonstrated that the anti-AD effects of Liubao tea are predominantly mediated by its characteristic bioactive components, including TBs, TFs, total tea pigments, and catechin monomers (EGC, EGCG, EC, ECG). These functional components collaboratively exert neuroprotective actions through three core intersecting mechanisms: modulation of ROS, MDA, and SOD to alleviate oxidative stress, regulation of IL-6 and TNF-α to suppress neuro-inflammation, and reduction in Aβ deposition and cellular senescence, as evidenced by altered SA-β-Gal activity. Notably, oxidative stress and neuro-inflammation constitute tightly interconnected pathological cascades. Based on the current biomarker data, we cannot definitively determine which pathway serves as the primary driver of neuroprotection. Additional assessments of mitochondrial function, protein homeostasis, synaptic integrity, and neuronal survival markers will help clarify the upstream and downstream mechanisms in future investigations. The distinct neuroprotective differences observed among different aroma types of Liubao tea indicate that post-fermentation and aging processes profoundly reshape the compositional profiles of bioactive constituents, thereby leading to divergent protective efficacies against AD-like pathological lesions. Such component-dependent functional variations provide a mechanistic basis for understanding how the unique chemical complexity of aged Liubao tea contributes to its multi-target neuroprotective effects. Nevertheless, we hypothesize that whole Liubao tea extract may exert superior efficacy compared with individual purified compounds. Botanical extracts frequently exhibit stronger biological activity than single isolated constituents due to multi-pathway synergistic effects. While individual compounds tend to regulate only a single signaling cascade, complex mixtures can simultaneously modulate oxidative stress, neuro-inflammation, and Aβ-associated pathology.

## 4. Materials and Methods

### 4.1. Experimental Materials and Instruments

Liubao tea, a traditional dark tea variety produced from the fresh leaves of *Camellia sinensis* (L.) O. Kuntze, was used in the present study. A total of twelve sample batches (designated **S1**–**S12**) were provided and taxonomically authenticated by China Tea (Wuzhou) Co., Ltd. (Wuzhou, China) (Table 2). The tea samples represented various aging gradients (unaged, 1-year, 3-year, 5-year, 10-year, and 15-year) and two typical aroma types (Aged aroma and Betel nut aroma).

The reference standards of epigallocatechin (EGC), catechin (C), epigallocatechin gallate (EGCG), caffeine (CAF), epicatechin (EC), epicatechin gallate (ECG), and L-theanine were purchased from Chengdu Must Bio-Technology Co., Ltd. (Chengdu, China). Gallic Acid was obtained from Shanghai Yuanye Biotechnology Co., Ltd. (Shanghai, China). The reactive oxygen species (ROS) assay kit was purchased from Beyotime Biotechnology Co., Ltd. (Shanghai, China). The BCA Protein Assay Kit, SOD and MDA detection kits, as well as assay kits for AChE activity, were all purchased from Edson Biotech Co., Ltd. (Shanghai, China). Modified double-antibody one-step sandwich Enzyme-linked immunosorbent assay (ELISA) kits for detecting zebrafish interleukin-6 (IL-6), tumor necrosis factor-α (TNF-α), and advanced glycation end products (AGEs) were obtained from Wankewi Biotech Co., Ltd. (Wuhan, China).

Except for D-Galactose (D-gal), donepezil, chromatographic grade methanol, acetonitrile, and formic acid which were supplied by Aladdin Biochemical Technology Co., Ltd. (Shanghai, China), other chemicals and reagents were obtained from Sinopharm Chemical Reagent Co., Ltd. (Shanghai, China). All reference standards, assay kits and reagents were of qualified experimental grade and used directly for subsequent phytochemical quantification and in vivo model experiments without further purification.

The main experimental instruments used in this study were as follows: a Zebrafish culture system (Z-A-D5, Shanghai Haisheng Biological Experimental Equipment Co., Ltd., Shanghai, China); fluorescence stereomicroscope (M165FC, Leica Microsystems GmbH, Wetzlar, Germany); illumination incubator (BXG-250, Medical Equipment Factory of Shanghai Boxun Industrial Co., Ltd., Shanghai, China); multifunctional microplate reader (SPARK, TECAN, Grödig, Austria); precision electronic balance (CP214, OHAUS, Corporation, Parsippany, NJ, USA); high-performance liquid chromatography (HPLC) system Model 2695 equipped with a 2489 PDA detector and Empower data processing system (Waters Corporation, Milford, MA, USA), coupled with a YMC-Pack ODS-AQ chromatography column (4.6 × 250 mm, 5 µm); and UV-visible spectrophotometer (UV-2600, Shimadzu Instruments (Suzhou) Co., Ltd., Suzhou, China). Larval locomotor activity was recorded using an automated video-tracking system (Viewpoint, Lyon, France).

### 4.2. Preparation of Liubao Tea Aqueous Extract for Chemical Quantification and Zebrafish Experiment

To ensure batch consistency, all Liubao tea samples were sourced from the same manufacturer following standardized production specifications. All extraction parameters, including solid–liquid ratio, extraction solvent, temperature, duration, and concentration procedures, were strictly controlled throughout the whole experiment.

Liubao tea aqueous extracts were prepared using a hot water extraction method for subsequent phytochemical quantification and zebrafish intervention experiments. A total of 25.0000 g of Liubao tea was added to 250 mL of boiling pure water and steeped at 90–100 °C for 20 min. After filtration through a three-layer 200-mesh filter cloth, the initial filtrate was collected. The tea residue was then re-extracted with an additional 250 mL of boiling water for another 20 min under the same conditions. The two filtrates were combined and diluted to a final volume of 500.00 mL to prepare the stock Liubao tea aqueous extract with a concentration of 50 mg·mL^−1^ (25.0000 g/500.00 mL = 0.05 g·mL^−1^ = 50 mg·mL^−1^). The prepared stock solution was used directly for subsequent phytochemical detection and zebrafish treatment after appropriate gradient dilution according to experimental requirements.

### 4.3. Analysis of Major Bioactive Constituents of Liubao Tea

Chromatographic separation was performed using a YMC-Pack ODS-AQ chromatography column (4.6 × 250 mm, 5 µm, YMC Co., Ltd., Kyoto, Japan). The mobile phase consisted of 0.1% formic acid aqueous solution (mobile phase A, aqueous phase) and methanol (mobile phase B, organic phase). This mobile phase system was ultimately selected for gradient elution (Figure S1, Supplementary Materials).

The column temperature was maintained at 30 °C, the flow rate was set to 0.8 mL/min, and the detection wavelength was 278 nm. The gradient program was strictly followed according to the actual experimental conditions as follows: 0–5 min, 10% B (equivalent to 90% A); 5–10 min, 20% B (equivalent to 80% A); 10–15 min, 32% B (equivalent to 68% A); 15–30 min, 32% B (equivalent to 68% A); and 30–35 min, 35% B (equivalent to 65% A).

Total tea polyphenol (TTP) content was determined according to the national standard GB/T 31740.2-2015 [21]. Total free amino acid (FAA) content was analyzed following GB/T 8314-2002 [22]. Proanthocyanidins (PC) were quantified by a vanillin-HCl colorimetric method using L-theanine as the standard via ultraviolet spectrophotometry [23]. Analysis of the principal tea pigments (TPs, containing theaflavin, TFs; thearubigin, TRs; and theabrownin, TBs) were conducted using a systems approach via ultraviolet spectrophotometry [24]. All detection procedures were carried out in strict accordance with corresponding standard specifications and established analysis protocols.

### 4.4. Experiments on Zebrafish

#### 4.4.1. Zebrafish Breeding Conditions

Wild-type zebrafish (AB strain) were obtained from the Zebrafish Joint Laboratory of Guangxi University of Chinese Medicine and were reared in a specialized zebrafish culture system under standardized conditions. The culture water was maintained at a pH of 6.50–7.50, a temperature of 28.5 °C, and a dissolved oxygen level of ≥6.00 mg·L^−1^. The zebrafish were fed three times daily, with brine shrimp (*Artemia salina*) provided twice and supplementary feed once, and they were acclimatized to a photoperiod of 14 h of light and 10 h of darkness.

#### 4.4.2. Establishment of Zebrafish AD-like Model

The establishment of the zebrafish AD-like model was based on previously published studies [25]. Three days post-fertilization (3 dpf) larval zebrafish were randomly transferred to six-well cell culture plates at a density of approximately 20 larvae per well. The larvae were continuously treated with 50 mM D-Gal from 3 to 6 dpf to successfully construct the zebrafish AD pathological model.

#### 4.4.3. Co-Treating Liubao Tea Aqueous Extract with D-Gal

Prior to the anti-AD experiment, an acute toxicity test was conducted to determine the safe working concentration of Liubao tea extract and to eliminate potential interference from toxic stress on the subsequent neuroprotective evaluation. LC_50_ represents the concentration causing 50% mortality of zebrafish larvae under the specified exposure conditions. Zebrafish larvae were exposed to concentrations of 1250, 625, 312.5, 156.25, and 78.125 μg·mL^−1^ from 3 to 6 dpf for the acute toxicity test. The LC_50_ value was calculated to be 774.76 μg·mL^−1^, (Figure S2, Supplementary Data). Therefore, 500 μg·mL^−1^ of Liubao tea extract (below the LC_50_ threshold) was selected for the subsequent anti-AD activity evaluation.

Donepezil, a classic acetylcholinesterase (AChE) inhibitor, was used as the positive control drug in this experiment. The specific treatment regimens for each group were as follows: the blank control group received neither D-Gal nor Liubao tea extract and was routinely cultured in standard embryo water; the AD-like model group was continuously exposed to 50 mM D-Gal alone from 3 to 6 dpf; the Liubao tea treatment group was co-treated with 50 mM D-Gal and 500 μg·mL^−1^ Liubao tea extract; and the positive control group was co-exposed to 50 mM D-Gal and 4.0 µM donepezil during the same experimental period. After continuous treatment, 10 larvae from each group were randomly selected for subsequent image capture and related index measurements.

### 4.5. Zebrafish Sample Collection and Test

#### 4.5.1. Behavioral Analysis of Zebrafish Larvae

At 3 dpf, 10 larvae were randomly selected from each group and individually placed into the wells of a 96-well plate, with 200 μL of the corresponding treatment solution added to each well. An acclimation period consisting of a single cycle of 5 min of light followed by 5 min of darkness was implemented to allow the larvae to adapt to the detection environment. The behavioral test included three consecutive cycles of 5 min of light and 5 min of darkness alternation. Changes in the average swimming speed and total swimming distance of zebrafish larvae in response to light–dark transitions were recorded and quantitatively analyzed to objectively evaluate the larvae’s behavioral responsiveness and sensorimotor function under light–dark stimuli.

#### 4.5.2. Determination of Aβ Deposition

The study involved continuous extract treatment in a zebrafish model to evaluate neuroprotective effects. After three days of continuous treatment with D-galactose and Liubao tea extract, zebrafish larvae from each group were rinsed twice with standard embryo water to remove residual treatment solution. They were then incubated in 0.3% Thioflavin S staining solution for 30 min in the dark at a constant temperature of 25 °C, followed by three washes with phosphate-buffered saline (PBS) to remove excess staining reagent. After staining, larval samples were imaged under the green fluorescence channel with excitation at 485 nm and emission at 525 nm. Clear top-view images of each larval head region were acquired using a fluorescence stereomicroscope, and the area and fluorescence intensity of Aβ plaques deposited in the head region were quantitatively analyzed using ImageJ software (Version 1.54p National Institutes of Health, Bethesda, MD, USA)).

#### 4.5.3. Determination of Senescence-Associated β-Galactosidase Activity

At 3 dpf, normally developed and healthy zebrafish larvae were selected, randomly assigned to experimental groups, and placed in 12-well plates at a density of 10 larvae per well. Three replicate wells were prepared for each group to ensure experimental reproducibility. The drug-containing embryo water in each well was renewed every 24 h throughout the experimental period. After 72 h of continuous treatment, the larvae were collected and fixed in a specialized fixative solution for 1 h, followed by being washed three times with PBS, each lasting 15 min to ensure thorough cleaning. Subsequently, the larvae were stained overnight with senescence-associated β-galactosidase (SA-β-Gal) staining solution under dark conditions. After staining, the residual staining solution was rinsed off with PBS, and the larvae were transferred to a Petri dish containing 3% sodium carboxymethylcellulose aqueous solution for fixation and positioning. The staining intensity of each larva was photographed and recorded under a stereomicroscope, and the staining results were quantitatively analyzed using Image-Pro Plus software6.0 (Media Cybernetics, Rockville, MD, USA).

#### 4.5.4. Determination of Reactive Oxygen Species Under a Fluorescence Microscope

After 3 dpf of continuous drug and modeling treatment, zebrafish larvae from each group were incubated with 10 μM DCFH-DA (2′,7′-dichlorodihydrofluorescein diacetate) working solution for 30 min in the dark at a constant temperature of 28 °C. Following incubation, the staining solution was discarded, and the larvae were thoroughly rinsed with PBS to remove any residual probe solution, then anesthetized with 0.16% tricaine for subsequent imaging. Lateral-view clear images of stained larvae were acquired using a fluorescence stereomicroscope for ROS fluorescence signal analysis. The fluorescence intensity of each sample was quantitatively measured using ImageJ software (Version 1.54p National Institutes of Health, Bethesda, MD, USA) to reflect the in vivo ROS accumulation level.

#### 4.5.5. Determination of SOD, MDA, AChE, IL-6 and TNF-α Levels

After 3 dpf of continuous drug treatment in the model, 150 zebrafish larvae from each experimental group were collected and pooled in centrifuge tubes. They were then homogenized in ice-cold normal saline at a tissue-to-liquid ratio of 1.0 g of tissue per 9.0 mL of normal saline to prepare the tissue homogenate. The homogenate was centrifuged at 3500 rpm for 15 min at 4 °C, and the supernatant was carefully collected. The total protein content of the supernatant was determined using a BCA Protein Assay Kit (Jiangsu Aidisheng Biotechnology Co., Ltd., Yancheng, China, Cat. No. ADS-W-SP002-48) to normalize the final detection results and ensure data accuracy. Levels of SOD, MDA, AChE, IL-6, and TNF-α were measured using corresponding commercial detection kits, following the manufacturer’s standard operating protocols strictly.

### 4.6. Statistical Analysis

All experimental tests in this study were independently repeated with at least three biological replicates to ensure the reliability and reproducibility of the data. Results are presented as means ± standard deviation (SD). Fixed numbers of zebrafish larvae were homogenized and pooled to form a single independent biological replicate. Statistical analyses were performed on pooled biological replicates rather than on individual larvae. All statistical calculations and data visualizations were conducted using IBM SPSS statistical software, version 26 (IBM Corporation, Armonk, NY, USA) and Origin 2022 plotting software. Differences among multiple experimental groups were assessed by one-way analysis of variance (one-way ANOVA), followed by Dunnett’s multiple comparison test. The Benjamini–Hochberg FDR multiple-testing correction was applied to minimize false-positive results arising from multiple comparisons. Statistical significance was defined as *p* < 0.05, and highly significant difference were defined as *p* < 0.01.

## 5. Conclusions

In summary, this study systematically elucidated the ameliorative effects of aged Liubao tea aqueous extract on D-galactose-induced pathological damage in a zebrafish AD-like model. Furthermore, this study revealed the aroma-dependent and component-targeted regulatory mechanisms underlying its anti-AD efficacy.

Both Aged aroma and Betel nut aroma Liubao teas exert synergistic neuroprotective effects, which are attributed to their abundant bioactive compounds, namely theabrownins, theaflavins, total tea pigments, and catechins. These functional ingredients collectively mitigate oxidative stress and suppress neuro-inflammation, thereby effectively alleviating abnormal Aβ deposition and aging-associated neuronal pathological phenotypes in the zebrafish AD-like model. A limitation of this study is the lack of a tea-only control group (i.e., Liubao tea extract administered to healthy zebrafish without AD induction). Consequently, we cannot fully distinguish the baseline physiological effects of the extract from its therapeutic effects under disease conditions. Several challenges currently hinder the clinical translation of Liubao tea products: (1) the complex chemical composition results in unidentified core active constituents and ambiguous target signaling pathways; (2) variability in raw material quality leads to insufficient batch-to-batch reproducibility; (3) data regarding pharmacokinetic profiles, bioavailability, and long-term safety remain limited; and (4) robust evidence from mammalian in vivo models and clinical trials is still lacking. Nevertheless, the present study provides fundamental theoretical support for developing Liubao tea as a promising natural candidate for anti-AD research. Prior to clinical evaluation in AD patients, further comprehensive investigations are required, including validation of neuroprotective efficacy in mammalian AD models; identification of key bioactive components and their underlying molecular mechanisms; evaluation of pharmacokinetic characteristics, bioavailability, and blood–brain barrier permeability of active constituents; systematic acute and chronic safety and toxicology assessments; and establishment of standardized quality control systems for raw tea materials and processed extracts to ensure stable and consistent quality. Subsequent research will combine behavioral assessments and gene knockout animal models to elucidate the direct modulation of TFs, TBs, and other pivotal components on Aβ aggregation, cellular senescence biomarkers, and neuro-inflammatory responses. Further investigation into upstream molecular signaling pathways will serve as a major direction for our future research.

## Figures and Tables

**Table 1:** Effects of Liubao tea on neuropathological and functional indexes in zebrafish AD-like model.

Neuropathological & Functional Indicators		Groups		Optimal Group
		Model	Liubao Tea (S1–S12)	
Motor Capability	swimming speed (mm·s^−1^)	0.250	1.435–7.294	**S12**
	total swimming distance (mm)	526.40	623.54–11,555.78	**S12**
AD Pathological Characteristics	SA-β-Gal activity	25.61	19.01–23.08	**S6**
	Aβ protein aggregation	23.38	13.70–20.07	**S6**
Oxidative Stress Reaction	ROS	1.69	0.75–1.42	**S1**
	SOD (U·mg prot^−1^)	175.09	213.42–432.44	**S1**
	MDA nmol·mg prot^−1^)	120.05	68.43–93.14	**S1**
Cholinergic Activity	AChE (nmol·mg prot^−1^)	2.27	1.32–1.53	**S6**
Inflammatory Mediators	IL-6 (pg·mg prot^−1^)	23.37	11.96–18.92	**S12**
	TNF-α (pg·mg prot^−1^)	28.52	16.24–24.48	**S12**

**Table 2:** Designations of Liubao tea.

Aroma Type	ID	Aging Years	Batch No.
Aged aroma (Chen Xiang)	**S1**	0	20230426
	**S2**	1	20230426
	**S3**	3	20230426
	**S4**	5	20230426
	**S5**	10	20230426
	**S6**	15	20230426
Betel nut aroma (Binglang Xiang)	**S7**	0	20230426
	**S8**	1	20230426
	**S9**	3	20230426
	**S10**	5	20230426
	**S11**	10	20230426
	**S12**	15	20230426

## Data Availability

The original contributions presented in the study are included in the article and Supplementary Materials, further inquiries can be directed to the corresponding authors.

## Supplementary Materials

The following supporting information can be downloaded at: https://www.mdpi.com/article/10.3390/ph19091336/s1, Figure S1: Effect of mobile phase composition on the chromatographic separation. (a) methanol with 0.1% formic acid aqueous solution; (b) methanol with water; (c) acetonitrile with 0.1% formic acid aqueous solution; (d) acetonitrile with water.; Figure S2: Dose-toxicity curve of Liubao tea aqueous extract on zebrafish larvae; Table S1: The validation data of the High-performance liquid chromatography (RSD%, n = 6); Figure S3: The chromatograms of the Liubao tea extract (S7, a) and standard (b); Figure S4: The chromatograms of the aged aroma Liubao tea extract; Figure S5: The chromatograms of the betel nut aroma Liubao tea extract; Table S2: Content (mg/g) of catechins and caffeine in Liubao tea water extract (n = 3); Table S3: Content (mg/g) of other characteristic chemical compositions in Liubao tea water extract (n = 3); Figure S6: Representative photographs (A) and staining images (B) illustrating the ameliorative effects of Liubao tea on AD-like pathological changes (neuronal senescence and Aβ amyloid deposition) in zebrafish; Figure S7: Representative staining images showing the ameliorative effect of Liubao tea on ROS oxidative stress in zebrafish; Table S4: Data from Spearman correlation analysis between Liubao tea components (S1−S12) and Alzheimer’s disease-related pharmacodynamic indexes; Table S5: Benjamini–Hochberg FDR-adjusted *p* values derived from Spearman correlation analysis between bioactive components of Liubao tea (S1−S12) and pharmacodynamic indices associated with Alzheimer’s disease.
