# Supplementary material for: Potential Ameliorative Effect of Aged Liubao Tea Aqueous Extract on D-Galactose-Induced Pathological Damage in an Alzheimer’s Disease Zebrafish Model
Source: Pharmaceuticals (Basel). 2026 Aug 24;19(9):1336. doi: 10.3390/ph19091336 (PMC13610899; doi:10.3390/ph19091336)
Supplement: Supplementary file 1 [file pharmaceuticals-19-01336-s001.zip › pharmaceuticals-4444162-Supplementary.pdf]

Potential Ameliorative Effect of Aged Liubao Tea Aqueous Extract on D-Galactose-Induced Pathological Damage in an Alzheimer's Disease Zebrafish Model

Shini Zhang [1], Xiao-Li Luo [1,2], Hangxing Yang [1], Ganghua Huang [3], Jing Leng [4,*] and Tao Yang [1,*]

[1] School of Pharmaceutical Sciences, Guangxi University of Chinese Medicine, Nanning 530200, China; shiniz@163.com (S.Z.); lxiaoli2017@126.com (X.-L.L.)
[2] Teaching and Experimental Training Centre, Guangxi University of Chinese Medicine, Nanning 530200, China
[3] China Tea (Wuzhou) Co., Ltd., Wuzhou 543000, China
[4] Guangxi Key Lab High Incidence Infect Dis Integrat, Guangxi University of Chinese Medicine, Nanning 530200, China
* Correspondence: lj986771558@163.com (J.L.); yt01_1986@163.com (T.Y.)

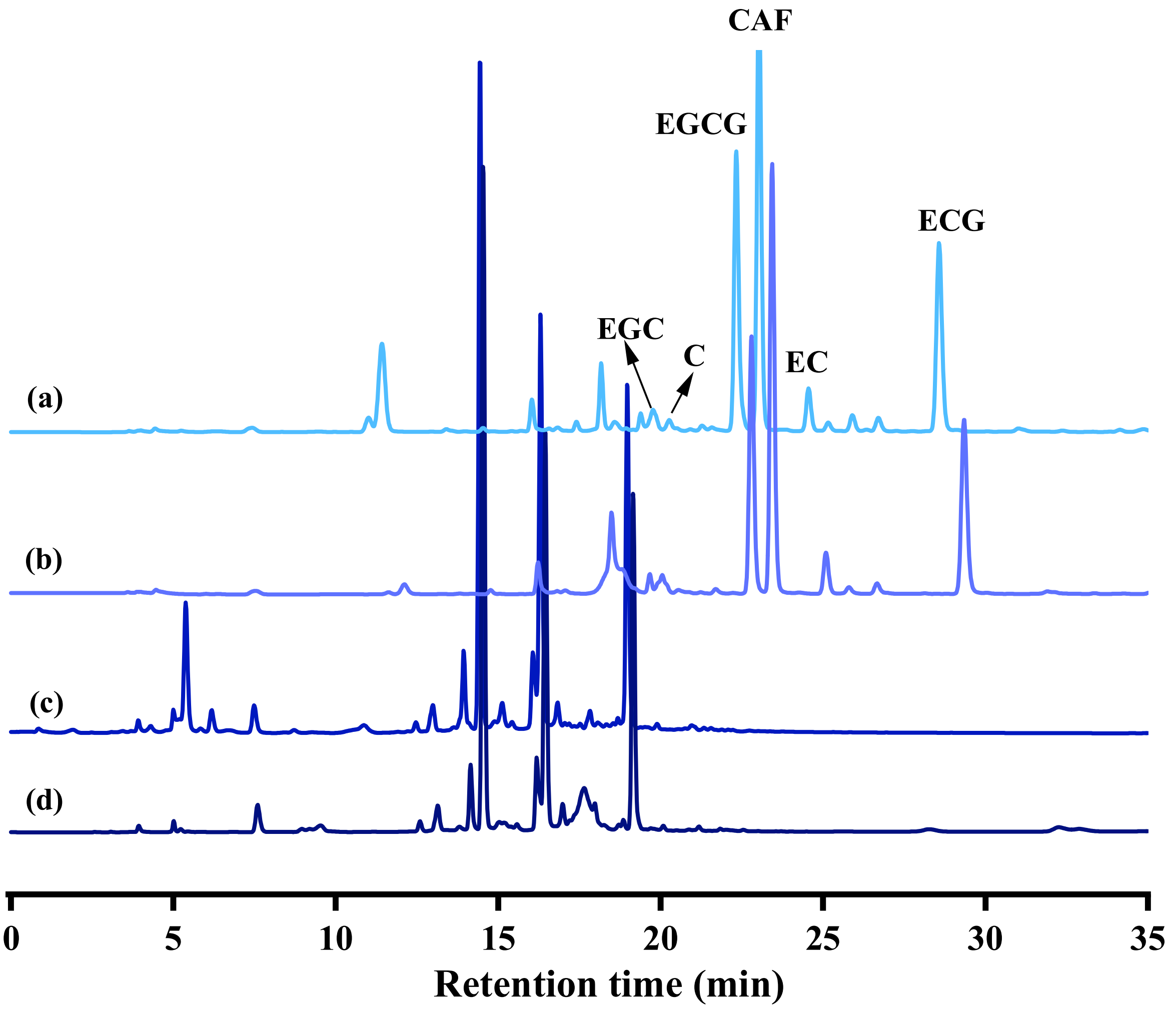

Figure. S1 Effect of mobile phase composition on the chromatographic separation. (a) methanol with 0.1% formic acid aqueous solution; (b) methanol with water; (c) acetonitrile with 0.1% formic acid aqueous solution; (d) acetonitrile with water.

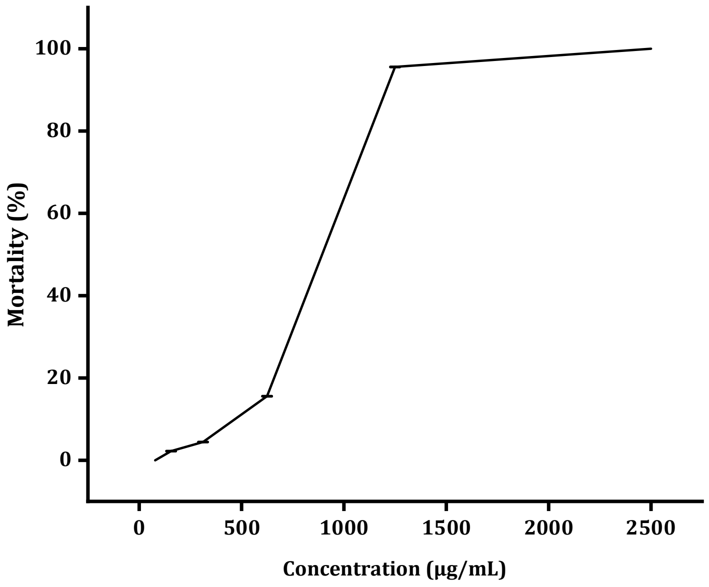

Figure. S2 Dose-toxicity curve of Liubao tea aqueous extract on zebrafish larvae.

Table S1 The validation data of the High-performance liquid chromatography (RSD%, n=6).

Analyte	Regression equations, R^2	Correlation Coefficients (R^2)	Precision	Repeatability	Stability	Recovery	Rs
EGC	Y=2.923251×10^{6}X-21578.109, 0.99941	0.001–1	0.05	1.95	2.81	1.77	1.51
C	Y=1.1791×10^{7}X-29452.62547, 0.99988	0.001–1	0.09	0.35	0.32	0.54	1.59
EGCG	Y=1.73196×10^{7}X-115156.8812, 0.99955	0.001–1	0.36	0.43	2.09	0.71	1.99
CAF	Y=3.55143×10^{7}X+174594.49074, 0.99967	0.001–1	0.10	0.37	0.49	0.41	1.63
EC	Y=8.487658×10^{6}X-7255.06744, 0.99999	0.001–1	0.17	1.01	1.12	0.59	2.06
ECG	Y=1.8538×10^{7}X-66701.54504, 0.99956	0.001–1	0.24	0.39	0.68	0.38	4.90

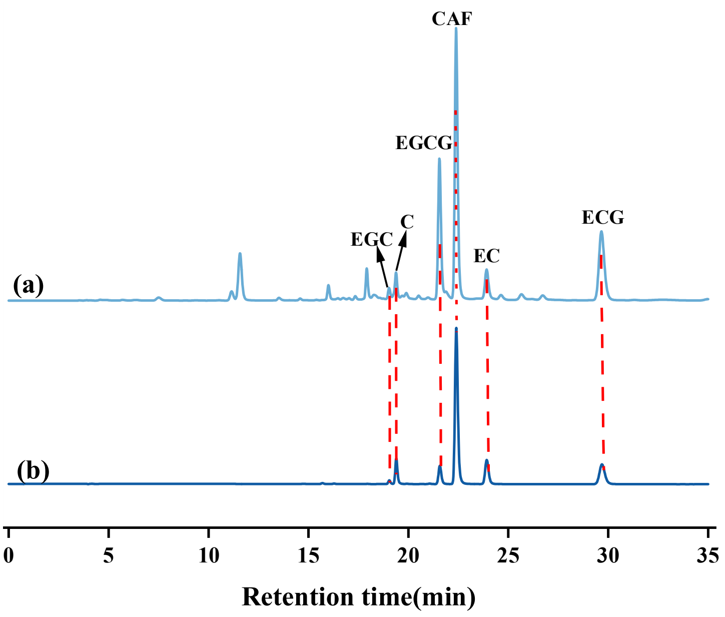

Figure. S3 The chromatograms of the Liubao tea extract (**S7**, a) and standard (b).

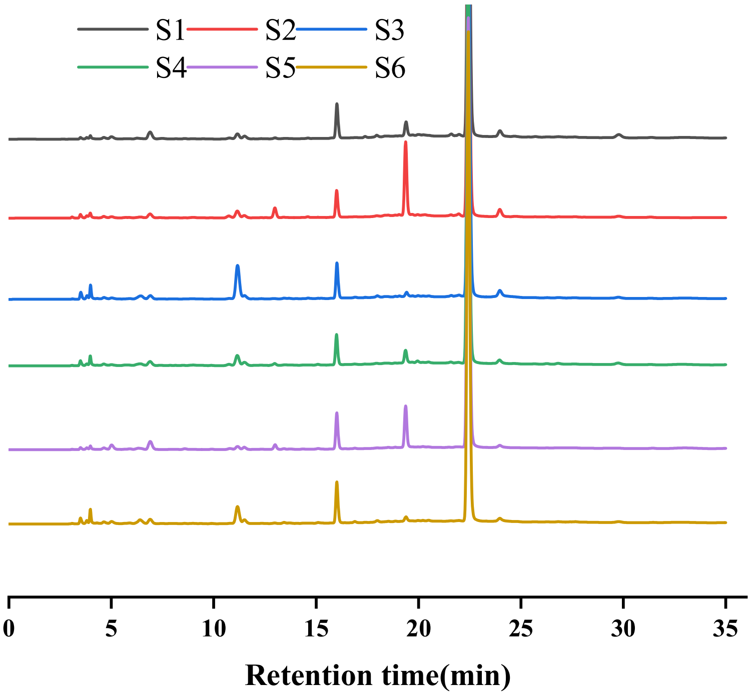

Figure. S4 The chromatograms of the aged aroma Liubao tea extract.

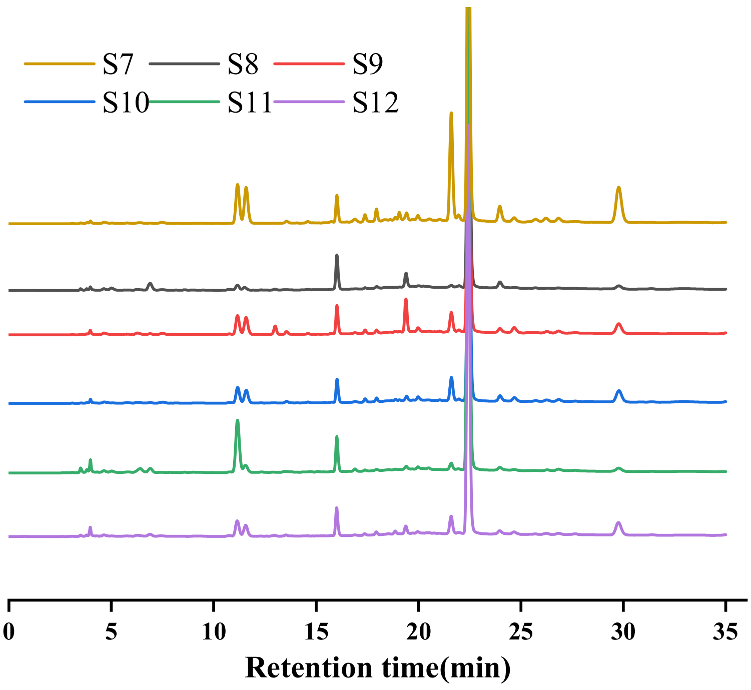

Figure. S5 The chromatograms of the betel nut aroma Liubao tea extract.

Table S2 Content (mg/g) of catechins and caffeine in Liubao tea water extract (n=3).

ID	CAF	Catechins					Total Catechins
		EGC	C	EGCG	EC	ECG	
S1	1.39±0.02	0.72±0.04	0.31±0.02	1.77±0.02	0.62±0.01	1.37±0.01	4.78 ±0.59
S2	1.25±0.01	0.10±0.00	0.62±0.01	0.05±0.01	0.17±0.04	0.03±0.01	0.96 ±0.24
S3	1.48±0.01	0.10±0.01	0.06±0.02	0.04±0.01	0.10±0.02	0.03±0.01	0.33 ±0.03
S4	1.50±0.01	0.07±0.00	0.13±0.04	0.04±0.01	0.05±0.04	/	0.29 ±0.04
S5	1.45±0.02	0.05±0.00	0.33±0.01	0.04±0.01	0.05±0.04	/	0.45 ±0.14
S6	1.60±0.01	0.06±0.04	0.06±0.02	0.04±0.02	0.05±0.01	0.02±0.01	0.23 ±0.02
S7	1.44±0.01	0.43±0.01	0.11±0.00	0.40±0.01	0.27±0.01	0.19±0.01	1.40 ±0.14
S8	1.48±0.01	0.12±0.03	0.15±0.02	0.04±0.01	0.10±0.01	/	0.41 ±0.05
S9	1.36±0.00	0.11±0.01	0.33±0.01	0.08±0.01	0.07±0.06	0.04±0.01	0.62 ±0.12
S10	1.41±0.04	0.14±0.01	0.07±0.002	0.17±0.04	0.120±0.06	0.11±0.01	0.62 ±0.04
S11	1.24±0.03	0.06±0.01	0.04±0.01	0.05±0.01	0.04±0.01	0.03±0.01	0.22 ±0.01
S12	1.35±0.01	0.08±0.010	0.08±0.001	0.15±0.04	0.07±0.02	0.18±0.02	0.49 ±0.03

/ The content was below the detection limit and could not be accurately quantified.

Table S3 Content (mg/g) of other characteristic chemical compositions in Liubao tea water extract(n=3).

ID	TTP	PC	FAA	tea pigment			TPs
				TFs	TRs	TBs	
S1	1.14±0.05	0.09±0.01	0.09±0.01	0.59±0.05	47.56±0.64	24.83±0.77	72.98±1.22
S2	0.47±0.01	0.17±0.03	0.04±0.00	0.80±0.06	13.50±4.78	147.73±6.34	162.03±1.63
S3	0.61±0.02	0.19±0.00	0.05±0.01	0.41±0.02	3.88±0.38	176.58±0.53	180.86±0.71
S4	0.96±0.02	0.21±0.01	0.05±0.01	0.35±0.01	3.19±1.37	109.80±0.69	113.33±1.02
S5	0.61±0.02	0.14±0.01	0.04±0.01	0.41±0.01	2.10±2.80	161.65±1.10	164.15±3.79
S6	0.87±0.02	0.09±0.02	0.04±0.02	0.72±0.10	19.95±2.32	165.37±2.30	186.05±2.67
S7	0.96±0.01	0.10±0.00	0.07±0.01	0.42±0.02	16.43±1.37	38.07±0.85	54.91±1.03

**S8**	0.77±0.02	0.10±0.01	0.05±0.01	0.44±0.01	3.79±0.86	94.36±1.07	98.59±0.57
**S9**	0.56±0.01	0.14±0.01	0.04±0.01	0.80±0.07	19.75±5.80	84.74±5.23	105.29±0.80
**S10**	0.65±0.01	0.13±0.01	0.04±0.01	0.77±0.06	22.85±1.42	83.63±2.18	107.25±2.49
**S11**	0.76±0.03	0.23±0.01	0.04±0.01	0.42±0.02	13.49±1.12	83.59±1.58	97.50±0.57
**S12**	0.52±0.01	0.12±0.02	0.04±0.02	0.67±0.02	16.98±2.03	120.77±1.64	138.62±0.28

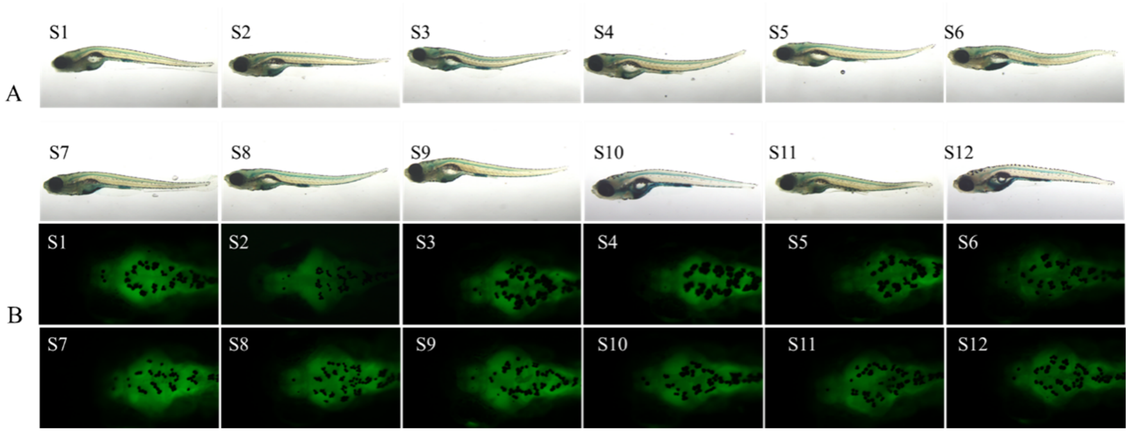

Figure. S6 Representative photographs (A) and staining images (B) illustrating the ameliorative effects of Liubao tea on AD-like pathological changes (neuronal senescence and Aβ amyloid deposition) in zebrafish.

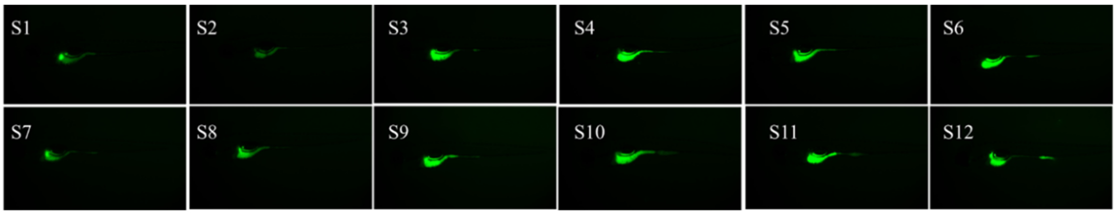

Figure. S7 Representative staining images showing the ameliorative effect of Liubao tea on ROS oxidative stress in zebrafish.

Table S4 Data from Spearman correlation analysis between Liubao tea components (**S1**–**S12**) and Alzheimer's disease-related pharmacodynamic indexes.

	EGC	C	EGCG	EC	ECG	TCs	CAF	TTP	PC	FAA	TFs	TRs	TBs	TPs
**SS**	0.07 (0.83)	0.14 (0.66)	0.13 (0.68)	0.05 (0.88)	0.11 (0.74)	0.03 (0.91)	-0.29 (0.37)	-0.15 (0.65)	-0.08 (0.80)	-0.31 (0.32)	0.73 (0.01)	0.28 (0.38)	0.22 (0.48)	0.20 (0.54)
**TSD**	-0.15 (0.65)	0.14 (0.66)	-0.14 (0.66)	-0.15 (0.63)	-0.15 (0.63)	-0.08 (0.81)	-0.15 (0.65)	-0.37 (0.24)	-0.08 (0.80)	-0.48 (0.11)	0.62 (0.03)	0.07 (0.83)	0.43 (0.17)	0.39 (0.21)
**Aβ**	0.62 (0.03)	0.01 (0.98)	0.69 (0.01)	0.34 (0.28)	0.60 (0.04)	0.36 (0.25)	-0.45 (0.14)	0.78 (0.00)	0.08 (0.80)	0.29 (0.35)	0.09 (0.78)	0.27 (0.39)	-0.92 (0.00)	-0.94 (0.00)
**SA-β-Gal**	0.52 (0.08)	0.01 (0.97)	0.34 (0.28)	0.41 (0.19)	0.20 (0.52)	0.18 (0.57)	-0.18 (0.57)	0.75 (0.01)	0.15 (0.63)	0.53 (0.08)	-0.29 (0.35)	-0.15 (0.65)	-0.71 (0.01)	-0.81 (0.00)
**ROS**	-0.32 (0.31)	-0.27 (0.39)	-0.06 (0.86)	-0.58 (0.05)	-0.06 (0.84)	-0.39 (0.21)	-0.24 (0.44)	-0.08 (0.80)	0.11 (0.73)	-0.62 (0.03)	0.17 (0.59)	-0.07 (0.83)	-0.06 (0.86)	-0.07 (0.83)
**SOD**	0.35 (0.27)	0.40 (0.20)	0.06 (0.86)	0.61 (0.04)	0.07 (0.83)	0.47 (0.12)	0.26 (0.42)	0.08 (0.80)	-0.21 (0.51)	0.69 (0.01)	-0.16 (0.62)	0.04 (0.90)	0.04 (0.90)	0.01 (0.97)
**MDA**	-0.90 (0.00)	-0.26 (0.42)	-0.54 (0.07)	-0.94 (0.00)	-0.41 (0.19)	-0.67 (0.02)	-0.13 (0.68)	-0.62 (0.03)	0.41 (0.18)	-0.74 (0.01)	-0.13 (0.68)	-0.38 (0.22)	0.45 (0.14)	0.45 (0.14)
**AChE**	-0.40 (0.32)	-0.21 (0.61)	-0.43 (0.29)	-0.05 (0.91)	-0.47 (0.24)	-0.21 (0.61)	0.71 (0.05)	-0.52 (0.18)	-0.55 (0.16)	0.12 (0.78)	-0.19 (0.65)	0.02 (0.96)	0.57 (0.14)	0.76 (0.03)
**IL-6**	-0.40 (0.32)	0.21 (0.61)	-0.81 (0.01)	-0.36 (0.39)	-0.85 (0.01)	-0.38 (0.35)	0.38 (0.35)	-0.26 (0.53)	0.69 (0.06)	0.48 (0.23)	-0.79 (0.02)	-0.83 (0.01)	0.17 (0.69)	0.05 (0.91)
**TNF-α**	-0.45 (0.26)	0.17 (0.69)	-0.76 (0.03)	-0.21 (0.61)	-0.74 (0.03)	-0.17 (0.69)	0.57 (0.14)	-0.50 (0.21)	0.07 (0.87)	0.33 (0.42)	-0.33 (0.42)	-0.48 (0.23)	0.64 (0.09)	0.55 (0.16)

Note: Values in parentheses represent Pearson correlation coefficients.

Table S5 Benjamini–Hochberg FDR-adjusted P values derived from Spearman correlation analysis between bioactive components of Liubao tea (**S1**–**S12**) and pharmacodynamic indices associated with Alzheimer's disease.

	EGC	C	EGCG	EC	ECG	TCs	CAF	TTP	PC	FAA	TFs	TRs	TBs	TPs
**SS**	0.918	0.868	0.868	0.935	0.909	0.938	0.705	0.868	0.918	0.657	0.112	0.717	0.82	0.868
**TSD**	0.868	0.868	0.868	0.868	0.868	0.918	0.868	0.595	0.918	0.454	0.212	0.918	0.546	0.572
**Aβ**	0.212	0.983	0.149	0.624	0.227	0.6	0.486	0.066	0.918	0.69	0.918	0.717	0.0009	0.0003
**SA-β-Gal**	0.366	0.972	0.624	0.572	0.859	0.868	0.868	0.112	0.868	0.343	0.69	0.868	0.131	0.0420*
**ROS**	0.657	0.717	0.927	0.254	0.927	0.572	0.767	0.918	0.906	0.212	0.868	0.918	0.927	0.918
**SOD**	0.619	0.572	0.927	0.212	0.918	0.486	0.735	0.918	0.849	0.153	0.868	0.938	0.938	0.972
**MDA**	0.002	0.735	0.341	0.000	0.572	0.162	0.868	0.212	0.572	0.112	0.868	0.586	0.486	0.486
**AChE**	0.657	0.868	0.628	0.938	0.6	0.868	0.254	0.572	0.536	0.918	0.868	0.972	0.486	0.212
**IL-6**	0.657	0.868	0.153	0.717	0.115	0.69	0.69	0.861	0.288	0.595	0.189	0.131	0.868	0.938
**TNF-α**	0.617	0.868	0.212	0.868	0.212	0.868	0.486	0.572	0.927	0.735	0.735	0.595	0.366	0.536
